# Virus-like nanoparticles as a theranostic platform for cancer

**DOI:** 10.3389/fbioe.2022.1106767

**Published:** 2023-01-12

**Authors:** Kyeong Rok Kim, Ae Sol Lee, Su Min Kim, Hye Ryoung Heo, Chang Sup Kim

**Affiliations:** ^1^ Graduate School of Biochemistry, Yeungnam University, Gyeongsan, South Korea; ^2^ Senotherapy-Based Metabolic Disease Control Research Center, Yeungnam University, Gyeongsan, South Korea; ^3^ School of Chemistry and Biochemistry, Yeungnam University, Gyeongsan, South Korea

**Keywords:** virus-like particle, drug delivery, bio-imaging, theragnosis, cancer

## Abstract

Virus-like nanoparticles (VLPs) are natural polymer-based nanomaterials that mimic viral structures through the hierarchical assembly of viral coat proteins, while lacking viral genomes. VLPs have received enormous attention in a wide range of nanotechnology-based medical diagnostics and therapies, including cancer therapy, imaging, and theranostics. VLPs are biocompatible and biodegradable and have a uniform structure and controllable assembly. They can encapsulate a wide range of therapeutic and diagnostic agents, and can be genetically or chemically modified. These properties have led to sophisticated multifunctional theranostic platforms. This article reviews the current progress in developing and applying engineered VLPs for molecular imaging, drug delivery, and multifunctional theranostics in cancer research.

## 1 Introduction

A wide range of liposome, synthetic and natural polymer, and inorganic nanoparticle (NP)-based carriers have been developed for tumor imaging and therapy (Malam et al., 2009; Ma et al., 2012; Beatty and Lewis, 2019). Properties of clinically effective carriers include efficient delivery, minimal toxicity, biocompatibility, and biodegradability. However, the use of synthetic carriers containing synthetic polymer-based NPs, liposomes, or metal-based NPs is limited due to their low stability, structural heterogeneity, potential immunogenicity, high toxicity, and off-target activity (Vabbilisetty and Sun, 2014; Cappellini et al., 2018; He and Tang, 2018; Lai and Wong, 2018; Alavi and Hamidi, 2019; de Ruiter et al., 2019). These limitations necessitate the search for alternative protein-based NPs. Protein-based NPs have several advantages, including a suitable size, uniform structure, controllable assembly, biocompatibility, biodegradability, and ease of functionalization (Bhaskar and Lim, 2017; Steinmetz, 2019; Comas-Garcia et al., 2020). Examples of protein-based NPs include virus-like particles (VLPs), ferritin, heat shock proteins, and vaults.

Considering that VLPs possess critical properties required for use in biomedical applications, such as water solubility, biocompatibility, and high cellular efficiency with minimal toxicity (Bhaskar and Lim, 2017; Beatty and Lewis, 2019; Comas-Garcia et al., 2020), they are an attractive option for use as a carrier platform in cancer therapy and diagnostics. VLPs from the bacteriophages Qubevirus durum (Qβ) and Emesvirus zinderi (MS2), tobacco mosaic virus (TMV), JC polyomavirus (JCPyV), human papillomavirus (HPV), hepatitis B virus (HBV), and cowpea chlorotic mottle virus (CCMV) have been used as carrier platforms in cancer research (Wang et al., 2016a; Barwal et al., 2016; Biabanikhankahdani et al., 2016; Chao et al., 2016; Kines et al., 2018; Pang et al., 2019a; Cai et al., 2020). Due to their hollow interior, VLPs can be loaded with various imaging agents and therapeutic molecules, including quantum dots (QDs), gadolinium (Gd), Gd-tetraazacyclododecane tetraacetic acid (Gd-DOTA), doxorubicin (DOX), small interfering RNA (siRNA), and proteins (Min et al., 2013; Bruckman et al., 2014; Li et al., 2015; Verwegen and Cornelissen, 2015; Barwal et al., 2016; Pitek et al., 2018; Pang et al., 2019b). VLPs can also be functionalized using genetic engineering and chemical ligation. In this article, we review the current research on VLPs with a uniform size distribution, internal cargo carrying capacity, and multi-functionality that are in development. We also discuss the current VLP-based cancer therapies and diagnostics.

## 2 Virus-like particles

VLPs are self-assembling protein-based capsular nanoparticles, 20–200 nm in size, composed of capsid proteins without genetic material. VLPs are non-infectious in nature, with the added benefit of being biocompatible and biodegradable. Furthermore, the size and morphology of VLPs can be controlled by manipulating the terminal amino acid of the capsid proteins and altering the pH of the buffer solution and the thermal conditions (Chen et al., 2001; Trifonova et al., 2017; Timmermans et al., 2018; Timmermans et al., 2022). Therefore, they have been utilized in various clinical applications, from disease diagnosis to treatment (Nooraei et al., 2021; Tariq et al., 2022).

VLPs are divided into enveloped VLPs (eVLPs) and non-enveloped VLPs (non-eVLPs) according to their structure (Nooraei et al., 2021; Mejía-Méndez et al., 2022; Tariq et al., 2022). eVLPs, composed of host cell-derived lipid membranes and glycoproteins, have a more complex structure than non-eVLPs, which are composed of single or multiple capsid proteins without lipid membranes. Due to their complicated structure, eVLPs are usually best expressed in eukaryotic systems (Cervera et al., 2013; Fluckiger et al., 2021). They are primarily used as vaccines as the carbohydrate antigens on viral glycoproteins can elicit immune responses (Dai et al., 2018; Carbaugh et al., 2019).

In contrast, non-eVLPs can be produced in both eukaryotic and prokaryotic expression systems owing to their relatively easy production and purification processes (Kushnir et al., 2012; Chen et al., 2020; Nooreai et al., 2021). Therefore, non-eVLPs have been mainly utilized as nanocarriers for therapeutics and diagnostics (Shan et al., 2018a; Shan et al., 2018b; Hu et al., 2019a; Hu and Steinmetz, 2020a; Bishnoi et al., 2021; Lu et al., 2021).

Depending on the morphology of the viral capsid, eVLPs are classified as isometric or helical structures (Aljabali et al., 2021). Non-eVLPs can be divided into isometric and rod-shaped filamentous structures (Parvez, 2020). The isometric structures of both eVLPs and non-eVLPs are spherical in shape with geometrically icosahedral symmetry (Pushko et al., 2013; Parvez, 2020). Icosahedral capsids can be characterized according to the triangulation number (T) proposed by Casper and Klug (Caspar and Klug, 1962). Icosahedral capsid proteins form substructures consisting of either five (pentamer) or six (hexamer) subunits. The T number indicates the number of capsid proteins required to envelop the virus, the degree of subdivision into pentamer and hexamer subunits, and the complexity of the icosahedral symmetry (Wilson, 2016), as the T number increases, the volume of the inner cavity of the VLPs also increased (Wilson, 2016; Sadre-Marandi and Das, 2018; Stone et al., 2019; Twarock and Luque, 2019).

VLPs can be functionalized with materials of interest, either chemically or genetically. Different types of VLPs have been developed by introducing various biological and chemical functional groups on their exterior and interior (Yoo et al., 2012; Sapsford et al., 2013; Thrane et al., 2015; Shan et al., 2018a). For example, cancer-targeting motifs or cancer antigens have been displayed on the external surface using genetic engineering to actively deliver VLPs into cancer or to induce an immune response (Wu et al., 2019; Li et al., 2021). Genetic conjugation has also been used to introduce non-natural amino acids with functional groups, such as azide or alkyne groups, in order to chemically conjugate functional materials onto the VLPs (Patel and Swartz, 2011). In addition to chemical functional groups, protein/peptide-based affinity systems have been used, including the Ni^2+^-His tag interaction, SpyTag-SpyCatcher interaction, and streptavidin-biotin interaction (Lim et al., 2013; Koho et al., 2015; Kim et al., 2019). Genetic conjugation has the advantage of introducing functional materials while minimizing the denaturation of VLP capsid proteins. However, this method has the disadvantage of causing misfolding of the VLP capsid protein (Mateu, 2011; Brune et al., 2016; Plateau et al., 2017).

Various chemical reactions have also been used to functionalize therapeutic agents or target motifs on VLP capsid proteins, particularly the formation of amide and disulfide bonds (Sletten and Bertozzi, 2009; Biabanikhankahdani et al. 2018). However, chemical conjugation also has the disadvantage of producing heterogeneous forms due to the difficulty of site-specific conjugation, which might reduce reproducibility (Pattenden et al., 2005; Mascola and Montefiori, 2010). Therefore, a conjugation method appropriate for the capsid protein and material to be introduced for VLP functionalization must be used.

**FIGURE 1:**
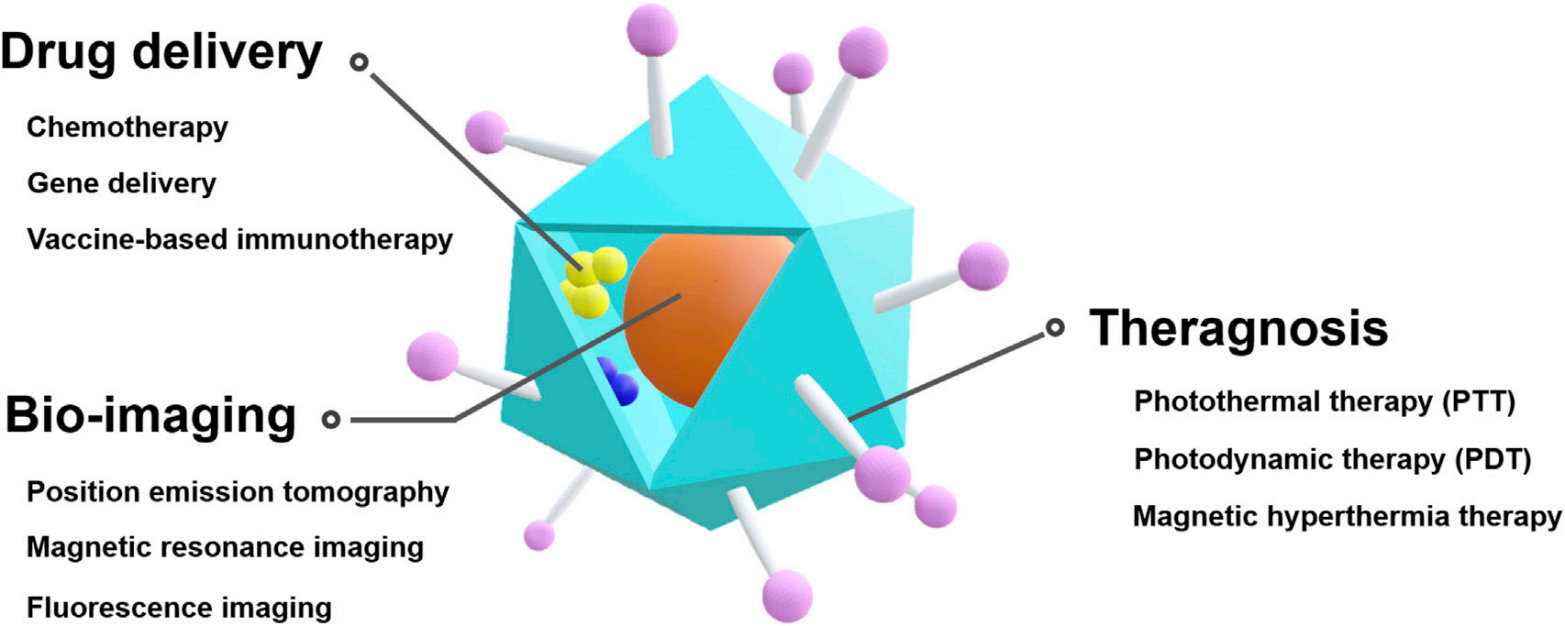
Schematic illustration of VLP-based theranostic platform for cancer therapy and diagnostics.

Considering that various characteristics, such as particle size, shape, and even functionality, can be controlled in VLPs, they are a promising platform for effective drug delivery, bio-imaging, and theragnosis (Figure 1). Specifically, VLPs have been studied as a functional nanocarrier in cancer treatment (Matsumura and Maeda, 1986; Liechty and Peppas, 2012; Wu, 2021).

## 3 Drug delivery

### 3.1 Chemotherapy

Chemotherapy is the most widely used method of cancer treatment, and it primarily uses doxorubicin (DOX), cisplatin, paclitaxel (PTX), and 5-fluorouracil (5-FU) to damage DNA, which leads to cell death (Tilsed et al., 2022). However, these drugs affect both cancer cells and normal cells (Sun et al., 2007). Drug carriers have been proposed to reduce the side effects of drugs (Gonda et al., 2019; Niculescu and Grumezescu, 2022; Tian et al., 2022). The size and shape of drug carriers are crucial for cancer targeting and cellular uptake (Albanese et al., 2012; Hoshyar et al., 2016). VLPs are good candidate drug carriers because they have various shapes and sizes that can be controlled by adjusting the type and number of subunits (Matsumura and Maeda, 1986). They also have excellent biocompatibility, a good size distribution, and a nano-sized capsular configuration.

Hepatitis B core protein virus-like particles (HBc VLPs) have been used to deliver DOX (Biabanikhankahdani et al., 2018). HBc VLPs have two distinct structures depending on their number of subunits; 240 subunits make 34 nm diameter particles with T = 4 symmetry and 180 subunits make 30 nm diameter particles with T = 3 symmetry (McGonigle et al., 2015). Folic acid (FA) was functionalized at the external lysine residues of HBc VLPs and DOX was conjugated to the surface-exposed carboxylate group using 1-ethyl-3-(3-dimethylaminopropyl) carbodiimide hydrochloride (EDC) and Sulfo-NHS. The DOX- and FA-functionalized HBc VLPs (DOX-FA-HBc VLPs) exhibited a higher cellular uptake by breast and colorectal cancer cells (HeLa and HT29) than normal cells (3T3 and CCD-112). Owing to their active targeting, DOX-FA-HBc VLPs had a lower half maximal inhibitory concentration (IC_50_) than free DOX in cancer cells, but a higher IC_50_ than free DOX in normal cells.

HBc VLPs have also been used as a carrier for the delivery of 5-fluorouracil-1-acetic acid (5-FA), a 5-FU derivative (Gan et al., 2020). The 5-FA molecules were conjugated to the external surface of HBc VLPs using EDC and Sulfo-NHS. The cell-penetrating peptide (CPP) of epithelial growth factor receptor (EGFR) was used for active targeting. EGFR is overexpressed in tumor cells and is involved in angiogenesis, invasion, and metastasis (Herbst, 2014). The CPP was co-synthesized with HBc capsid-binding peptide (nanoglue); these were then conjugated to the HBc VLPs using EDC and Sulfo-NHS. 5-FA- and CPP-conjugated HBc VLPs showed more internalization and cytotoxicity in A431 cells, which express considerably high levels of EGFR, than in HT29 or HeLa cells, indicating that the VLPs were delivered into the cells in an EFGR-dependent manner. Moreover, unlike free 5-FA, the 5-FA conjugated to HBc VLPs demonstrated a similar apoptotic activity to that of free 5-FU. pH-responsive functionalities have been introduced to control the release of chemotherapeutic agents from HBc VLP carriers in the slightly acidic tumor microenvironment. Polyacrylic acid (PAA) was introduced into HBc VLPs for the controlled release of DOX (Biabanikhankahdani et al., 2016). PAA can reversibly interact with DOX in a pH-dependent manner. DOX and polyacrylic acid (PAA) complexes were encapsulated in HBc VLPs using the disassembly and reassembly method. A pentadecapeptide containing the nanoglue was used and FA molecules were conjugated to the free Lys at the N-terminal end of the pentadecapetide bound on the HBc VLPs. The cumulative release of DOX from the HBc VLPs was significantly higher at pH 5.4 than that at pH 7.4. This result indicated that DOX release could be controlled in tumor and endosomal conditions. Owing to the FA-based active targeting, the HBc VLPs led to the accumulation of more DOX in colorectal cancer HT29 and Caco-2 cells than in normal CCD-112 cells. The HBc VLPs exhibited approximately a 5-fold lower IC_50_ in HT29 and Caco-2 cells and an approximately 2-fold higher IC_50_ in CCD-112 cells than that of free DOX.

In addition, the His tag has been utilized to develop HBc VLPs for the controlled release of DOX (Biabanikhankahdani et al., 2017). Depending on the pH, the His tag reversibly interacts with nitrilotriacetic acid (NTA) *via* Zn^2+^ (Bekele et al., 2022). NTA-DOX was non-covalently conjugated to His-tagged HBc VLPs. For active targeting, FA was conjugated to the HBc VLPs using EDC and Sulfo-NHS (DOX-NTA-FA-HBc VLPs). These HBc VLPs released significant levels of DOX at pH 5.4, whereas the drug was slowly released from the VLPs at pH 7.4. The DOX-NTA-FA-HBc VLPs showed higher cellular uptake in ovarian cancer OVCAR-3 cells than in normal 3T3 cells. These HBc VLPs also exhibited an approximately 3-fold lower IC_50_ in OVCAR-3 cells and an approximately 3-fold higher IC_50_ in 3T3 cells than that of free DOX.

The pH-dependent assembly and disassembly property of HBc VLPs has been used without introducing external motifs for the controlled release of DOX (Shan et al., 2018a). This strategy requires the encapsulation of DOX in the inner space of HBc VLPs. However, most anticancer chemotherapeutic agents are hydrophobic; therefore, the encapsulation efficiency of the drugs was low (Lu et al., 2007; Naderinezhad et al., 2017). Lipophilic NS5A peptides have been genetically introduced to the C-terminal ends of HBc capsid proteins to mitigate this limitation. In addition, RGD peptides have been inserted into the loop regions of HBc capsid proteins for active targeting. DOX was loaded into the inner space of the modified HBc VLPs (RGD-HBc-NS5A) using the disassembly and reassembly method in a pH-dependent manner. The DOX-loading capacity of RGD-HBC-NS5A was 2-fold higher than that of HBc VLPs without NS5A peptides. The pH-responsive properties of HBc VLPs enabled the controlled the release of DOX in the slightly acidic tumor microenvironment. The VLPs released 70% of DOX over 48 h at pH 5.0, whereas only 40% of DOX was released at pH 7.4. Furthermore, RGD-HBc-NS5A resulted in a 67% higher accumulation of DOX in cancer cells than did HBc-NS5A within 24 h.

In addition to HBc VLPs, several other VLPs have been used as carriers for the delivery of anticancer drugs. Physalis mottle virus (PhMV) VLPs have been used as a carrier to deliver DOX (Hu and Steinmetz, 2020a). PhMV VLPs have received substantial attention as a drug carrier platform because they have a long circulation half-life of ∼44 h, and ∼6% of the injected dose remains in the tumor site (Hu et al., 2019a). PhMV VLPs are 30 nm icosahedral particles with T = 3 symmetry, assembled by 180 coat proteins (Krishna et al., 1999). 6-maleomidocaproyl-hydrazone doxorubicin (DOX-EMCH) was prepared to contain an acid-sensitive hydrazine linker to release DOX in acidic conditions (Willner et al., 1993). The DOX-EMCH was loaded into the inner space of PhMV VLPs by a combination of the thio-maleimide reaction and π-π stacking interactions. The external surface of PhMV VLPs was modified with PEG to prevent non-specific cell uptake and improve biocompatibility (DOX-PhMV-PEG VLPs). Owing to their effective delivery and release of DOX in the slightly acidic tumor microenvironment, DOX-PhMV-PEG VLPs exhibited an approximately 3.4-fold higher therapeutic efficacy in tumor-bearing mice compared with that of free DOX.

**FIGURE 2:**
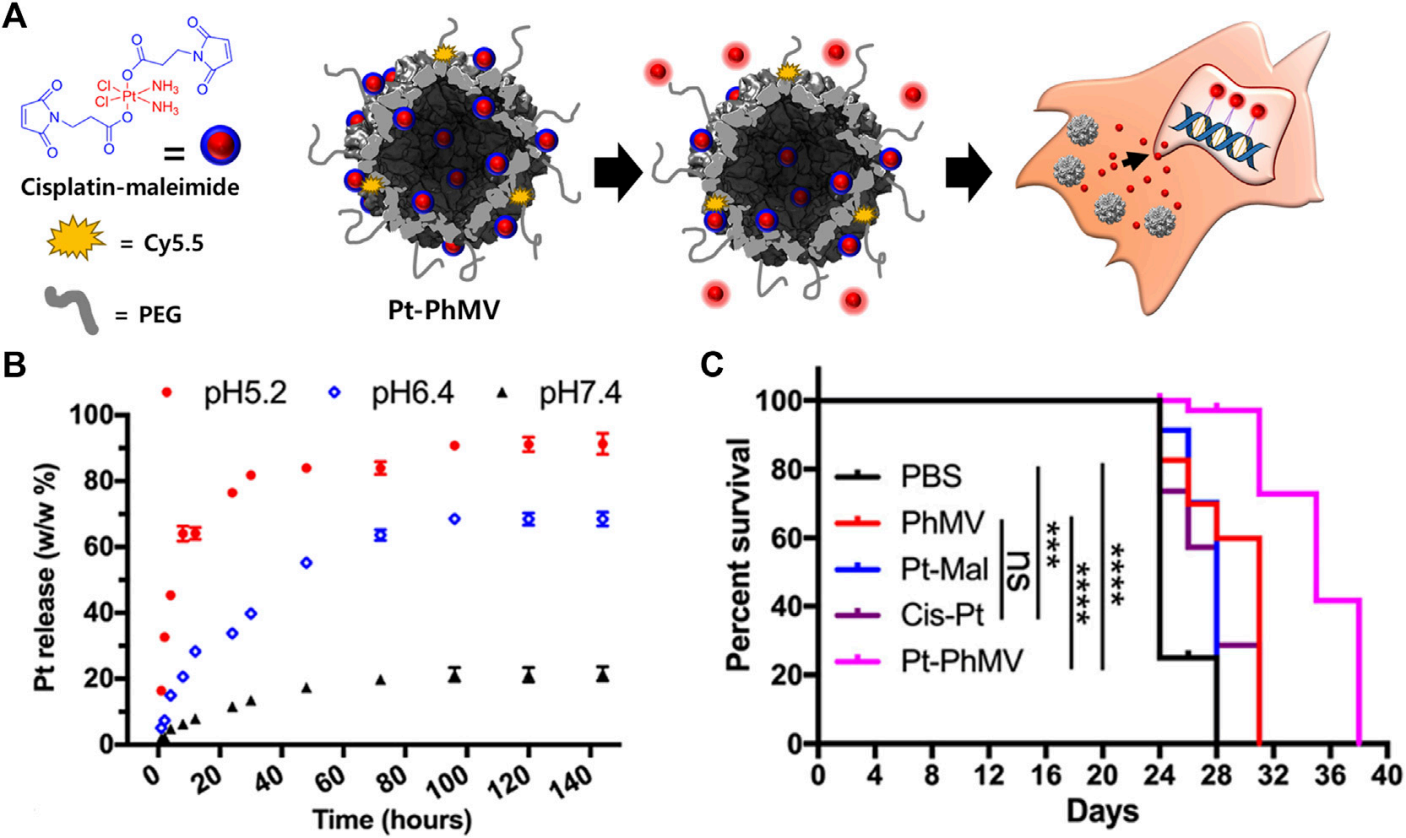
**(A)** Schematic illustration of synthesizing Pt-loaded and PEGylated PhMV VLPs (Pt-PhMV) for cancer therapy. **(B)** The pH-dependent controlled release profile of Pt from Pt-PhMV. **(C)** Survival of an athymic mouse model with MDA-MB-231 xenografts after treatment with Pt-PhMV. Reprinted with permission from Hu and Steinmetz (2020b). Copyright (2020) American Chemical Society.

PhMV VLPs have also been used to deliver a cisplatin prodrug containing platinum (Pt) IV (Hu and Steinmetz, 2020b) (Figure 2). A maleimide-functionalized cisplatin prodrug was conjugated to the internal cysteine residues of the PhMV coat protein *via* a thiol-maleimide reaction (Pt-PhMV). Cisplatin prodrugs are the most effective anti-cancer drugs for solid tumors, such as breast cancer. The pH-sensitive cisplatin might be reduced to a DNA-reactive Pt (II) complex in the acidic extracellular environment of a tumor. The outer surface of Pt-PhMVs was modified with polyethylene glycol (PEG) and cyanine (Cy) 5.5 to increase their biocompatibility and track them *in vivo*. Compared to free cisplatin and cisplatin-maleimide, these VLPs significantly prolonged the survival of cancer-affected mice by effectively inhibiting the growth of xenograft MDA-MB-231 breast tumors *in vivo*.

Foot-and-mouth disease virus-like particles (FMDV VLPs) have been proposed as a carrier for DOX (Yan et al., 2017). Considering that the surface of FMDV VLPs includes highly conserved arginine-glycine-aspartate (Arg-Gly-Asp; RGD) peptides that can act as a cancer-targeting motif, these VLPs enable active targeting without the need to introduce an external targeting motif. FMDV belongs to the Aphthovirus genus of the *Picornaviridae* family. The capsid consists of 60 copies of each of the four structural proteins (VP1 to VP4), which self-assemble into 30 nm icosahedral particles (Acharya et al., 1989). DOX was conjugated to the surface of FMDV VLPs using EDC and NHS. DOX-conjugated FMDV VLPs exhibited different release patterns of DOX depending on pH; the release rate increased as the pH became more acidic. This might be due to the denaturation of FMDV VLPs at a low pH. Owing to their active targeting, these VLPs exhibited a higher cellular uptake efficiency and cytotoxicity in HeLa cells than in normal F81 cells, and were less sensitive to F81 cells compared with free DOX. Therefore, FMDV VLPs induced more apoptosis in HeLa cells than in F81 cells.

Cowpea chlorotic mottle virus (CCMV) VLPs have been used to deliver DOX due to their high stability in acidic conditions (Barwal et al., 2016). CCMV is one of the *Bromoviridae* family of plant viruses and is composed of 180 capsid proteins. CCMV VLPs have a 30 nm icosahedral shell with T = 3 quasi-symmetry (Speir et al., 1995). The RNA-binding and β-hexamer-forming domains of the CCMV capsid proteins were genetically deleted. DOX was introduced to CCMV VLPs using two strategies. First, DOX was conjugated to the carboxylic groups of Glu and Asp of the CCMV capsid protein using EDC and NHS. Second, DOX was encapsulated by assembling CCMV capsid proteins around gold nanoparticles (AuNPs) that were conjugated to DOX using lipoic acid as a linker to improve the DOX-loading efficiency. In addition, FA was conjugated to CCMV VLPs using EDC-NHS chemistry to add cancer-targeting capability. FA-conjugated CCMV VLPs exhibited an approximately 2-fold higher cellular uptake in FR-overexpressing MCF-7 cells than that of CCMV VLPs without FA molecules. FA- and DOX-conjugated CCMV VLPs containing DOX-functionalized AuNPs showed higher cytotoxicity to MCF7 than VLPs without DOX-functionalized AuNPs.

*Salmonella typhimurium* bacteriophage P22 VLPs have been used to deliver DOX due to their large interior cavity (Kim et al., 2019). The P22 VLPs are 56 nm icosahedral particles with T = 7 symmetry, assembled by 420 coat proteins (Botstein et al., 1973). For active targeting, affibody (Afb) molecules were used against EGFR and human epidermal growth factor receptor 2 (HER2). The EFGR or HER2 Afb molecules were conjugated using the SpyTag/SpyCather system, in which SpyCatcher-fused Afb molecules were reacted with SpyTag peptide-fused P22 capsid proteins. DOX-EMCH was chemically conjugated to the interior of the SpyTag-displayed P22 VLPs. In contrats to wild P22 VLPs, HER2 Afb- or EFGR Afb-conjugated P22 VLPs exhibited high cellular uptake in MDA-MB-468 and SK-BR-3 cells that overexpressed EGFR and HER2 on their membranes, respectively. The modified P22 VLPs also showed high cytotoxicity to MDA-MB-648 and SK-BR-3 cells. However, they were less cytotoxic to MCF-7 breast cancer cells than free DOX. These results indicated that the modified P22 VLPs had high targeting ability.

### 3.2 Gene therapy

**FIGURE 3:**
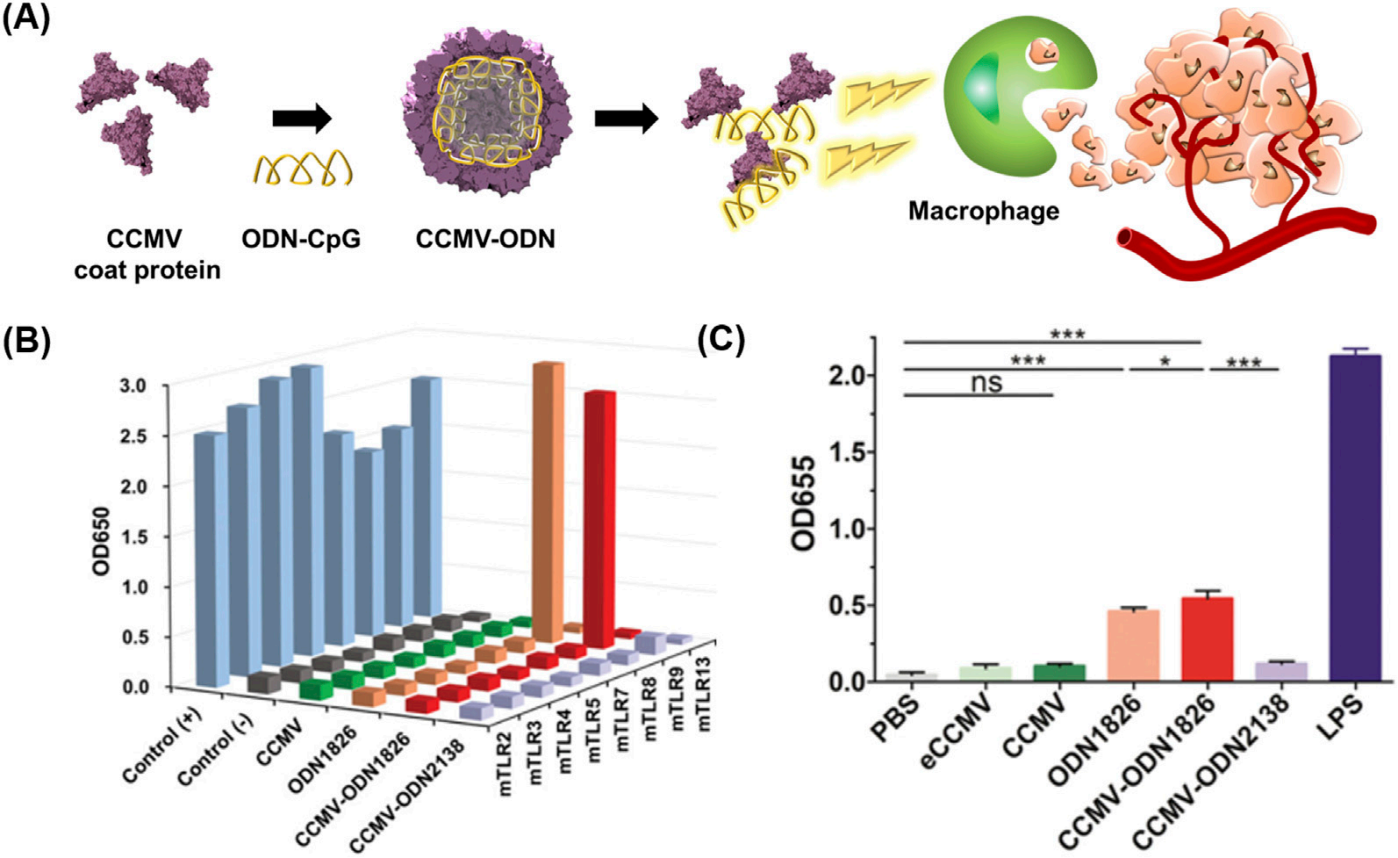
**(A)** Schematic diagram of preparing CCMV VLPs containing ODN 1826 (CCMV-ODN1826) for cancer therapy. **(B)** NF-κB activation ability of CCMV-ODN1826. **(C)** Stimulation of pattern recognition receptors by CCMV-ODN1826 in RAW-Blue cells. Reprinted with permission from Cai et al. (2020). Copyright (2020) Wiley-VCH GmbH.

Oligodeoxynucleotides (ODNs) with cytosine-guanine dinucleotide (CpG) motifs have been considered potent immunostimulatory drugs (Klinman, 2004; Hanagata, 2012; Chuang et al., 2014). Nevertheless, ODNs have an unfavorable pharmacokinetic profile and adverse effects (Zhang et al., 2012; Zhang et al., 2017; Zhang and Gao, 2017). VLPs have been proposed for the targeted delivery of ODNs to tumor-associated macrophages (TAMs) while minimizing the side effects of ODNs (Figure 3) (Cai et al., 2020).

Cowpea chlorotic mottle virus (CCMV) VLPs were used for the targeted delivery of ODN 1826, which induces the phagocytic activity of TAMs by activating the Toll-like receptor (TLR) 9 signaling pathway (Liu et al., 2019). ODN1826 was loaded in the inner space of CCMV VLPs using the disassembly and reassembly method, producing ODN1826-encapsulated VLPs (CCMV-ODN1826). CCMV-ODN1826 had a higher cellular uptake in TAMs than in murine subcutaneous colon cancer, and in comparison to free ODN 1826, they significantly enhanced the phagocytic activity of TAMs. Moreover, compared to free ODN 1826, CCMV-ODN1826 resulted in reduced tumor growth and prolonged survival in mouse models of colon cancer and melanoma.

CCMV VLPs have also been explored as a carrier for siRNAs (Lam and Steinmetz, 2019). CCMV VLPs were disassembled and reassembled in a pH- and salt-dependent manner to produce CCMV VLPs encapsulating siRNA for forkhead box transcription factor (FOXA1) as a therapeutic target (CCMV-siRNA). FOXA1 overexpression promotes tumor metastasis and invasion of prostate cancer, while inhibiting anticancer immune responses (Imamura et al., 2012; He et al., 2021). M-lycotoxin peptide L17E was chemically conjugated as a CPP to the outer surface of CCMV-siRNA. These VLPs led to the knockdown of FOXA1 mRNA levels in MCF7 cells, with approximately 50% of the effectiveness of lipofectamine.

Bacteriophage MS2 virus-like particles (MS2 VLPs) have been used to deliver microRNA (MiR-122) to target hepatocellular carcinoma (HCC) (Wang et al., 2016a). MS2 VLPs are 27 nm icosahedral particles with T = 3 symmetry (Valegard et al., 1990; Golmohammadi et al., 1993). The human immunodeficiency virus TAT peptide was genetically displayed on MS2 VLPs to enable them to penetrate cell membranes efficiently. Insulin-like factor 1 receptor and cyclin G1, which are associated with carcinogenesis (Musgrove et al., 1994; Hua et al., 2020), were downregulated by MiR-122-TAA-MS2 VLPs in HCC cell lines, including Hep3B, HepG2, and Huh7. The VLPs also induced apoptosis in the 3 cell lines, particularly in Hep3B and Huh7 cells. Furthermore, the tail vein-injected MiR-122-MS2 VLPs resulted in remarkably slower tumor growth than that by MS2 VLPs with non-target miRNAs in Hep3B-bearing BALB/c nude mice.

Cancer-specific motifs have been introduced for the active targeting of VLPs. HER2 Afb was genetically introduced on HBc VLPs to deliver siRNA for the Polo-like kinase 1 gene (siPLK1) (Suffian et al., 2018). PLK1 is overexpressed in several cancers, and is involved in cell division and the regulation of mitosis (Smith et al., 2017; de Cárcer et al., 2018). The siPLK1 was encapsulated in HER2 affibody-displayed HBc VLPs using disassembly and reassembly methods. The modified HBc VLPs showed enhanced cellular uptake compared to HBc VLPs without HER2 Afb in MDA-MB-468 and SKBR-3 cells. The HER2 affibody-displayed HBc VLPs also resulted in a reduced solid tumor mass in HER2-expressing intraperitoneal tumor mouse models compared to untreated group.

Neurotropic JC polyomavirus (JCPyV) VLPs have significant potential as a carrier for gene therapy in human glioblastoma because human glioblastoma cells are highly susceptible to the virus (Del Valle et al., 2001; Del Valle et al., 2002). These VLPs were used without the introduction of a cancer-specific motif to deliver a plasmid containing cancer-targeting genes into human glioblastoma (Chao et al., 2018). JCPyV VLPs consist of 360 copies of the VP1 coat protein arranged into 72 pentamers (Dinakar et al., 1986). The thymidine kinase suicide gene (tk) expression plasmid was encapsulated in JCPyV VLPs by co-transformation with a JCPyV VP1 expression plasmid in *E. coli*. When treated with intratumoral injection of the JCPy VLPs in combination with ganciclovir (GCV), the nude mouse model with orthotopic tumors exhibited prolonged survival by inhibiting cell growth of the human malignant glioblastoma U87 cells compared to GCV and JCPy VLPs without GCV. Furthermore, the tail vein-injected VLPs could deliver a suicide gene to implanted U87 cells *via* the circulatory system in this mouse model.

Combinations of chemo- and gene-therapeutic agents allow for cancer treatment with increased efficacy. HBc VLPs have been used as a carrier for simultaneous chemotherapy and gene therapy (Yang et al., 2020). The brain-targeting peptides TGN and RGD have been used for active targeting. Both the TGN and RGD peptides were genetically introduced to the HBc capsid protein. The dual-modified HBc VLPs were prepared by disassembling TGN-modified VLPs and RGD-modified VLPs and mixing them in equal proportions (TGN/RGD-HBc VLPs). PTX and siRNA for yes-associated protein YAP were serially encapsulated in TGN/RGD-HBc VLPs using the disassembly and reassembly method (PTX/siRNA@TGN/RGD-HBc VLPs). YAP is a transcriptional co-activator of the Hippo pathway, which plays a substantial role in the migration and invasion of glioma cells (Zhang et al., 2018a). PTX is a microtubule-associated cell replication inhibitor and has been demonstrated to reach the brain in small amount (Yardley, 2013; Zhu and Chen, 2019). The PTX/siRNA@TGN/RGD-HBc VLPs effectively targeted the brain and resulted in more tumor growth inhibition than PTX@TGN/RGD-HBc VLPs and saline in orthotopic U87-Luci tumor-bearing mice.

### 3.3 Vaccine-based immunotherapy

VLPs have been considered an attractive vaccine platform because they have a high density of repetitive antigenic epitopes on their surface that elicit humoral and cell-mediated responses (Tariq et al., 2022). To date, four VLP-based vaccines have been licensed and are commercially available (Dai et al., 2018; Mokoena et al., 2019). Following the success of these vaccines, additional VLP-based cancer vaccines have also been proposed and developed.

P22 VLPs displaying the B and T epitopes of ovalbumin (OVA_B_ peptide and OVA_T_ peptide) have been developed as therapeutic cancer vaccines (Li et al., 2021). The OVA_B_ and OVA_T_ peptides are tumor-specific neoantigens produced by somatic mutations in tumor cells and can stimulate the cytotoxic lymphocyte (CTL) response, resulting in a strong anti-tumor immune response (Zhang et al., 2021). Together with the adjuvant poly (I:C), OVA_B_-P22 VLPs induced a robust humoral immune response against the OVA_B_ antigen, and OVA_T_-P22 VLPs significantly inhibited tumor growth by activating tumor-specific CTL responses.

Rabbit hemorrhagic disease virus (RHDV) VLPs have been modified into a tumor vaccine by genetically fusing them with murine topoisomerase IIα (TopIIα) and survivin (Donaldson et al., 2017). RHDV VLPs are ∼40 nm icosahedral particles with T = 3 symmetry assembled by 90 dimers of the VP6 capsid protein (Bárcena et al., 2015). MC38-OVA tumor-bearing mice were vaccinated with TopIIα/survivin-RHDV VLPs conjugated to the adjuvant CpG ODN. These VLPs resulted in decreased tumor growth compared to PBS-treated group, thus demonstrating the efficacy of the VLP-based vaccine.

Several VLP-based cancer vaccines target cell surface glycoproteins. Mucin-1 (MUC1), a highly O-glycosylated glycoprotein, is overexpressed in various cancer cells. It plays a substantial role in cancer progression and development (Nath and Mukherjee, 2014; Gao et al., 2020). Therefore, as with protein antigens, the glycan chain of MUC1 can be a target for cancer immunotherapy. Qβ VLPs presenting tumor-associated MUC1 have been developed, and their efficacy as an anti-cancer vaccine have been demonstrated (MUC1-Qβ VLPs) (Wu et al., 2019). MUC1-Qβ VLPs together with the adjuvant monophosphosphoryl lipid A elicited high levels of anti-MUC1 IgG antibodies in MUC1 transgenic mice. Moreover, the antibodies produced strongly bound to MUC1-expressing melanoma B16-MUC1 cells, demonstrating the potential of MUC1-Qβ as an anti-cancer vaccine.

**FIGURE 4:**
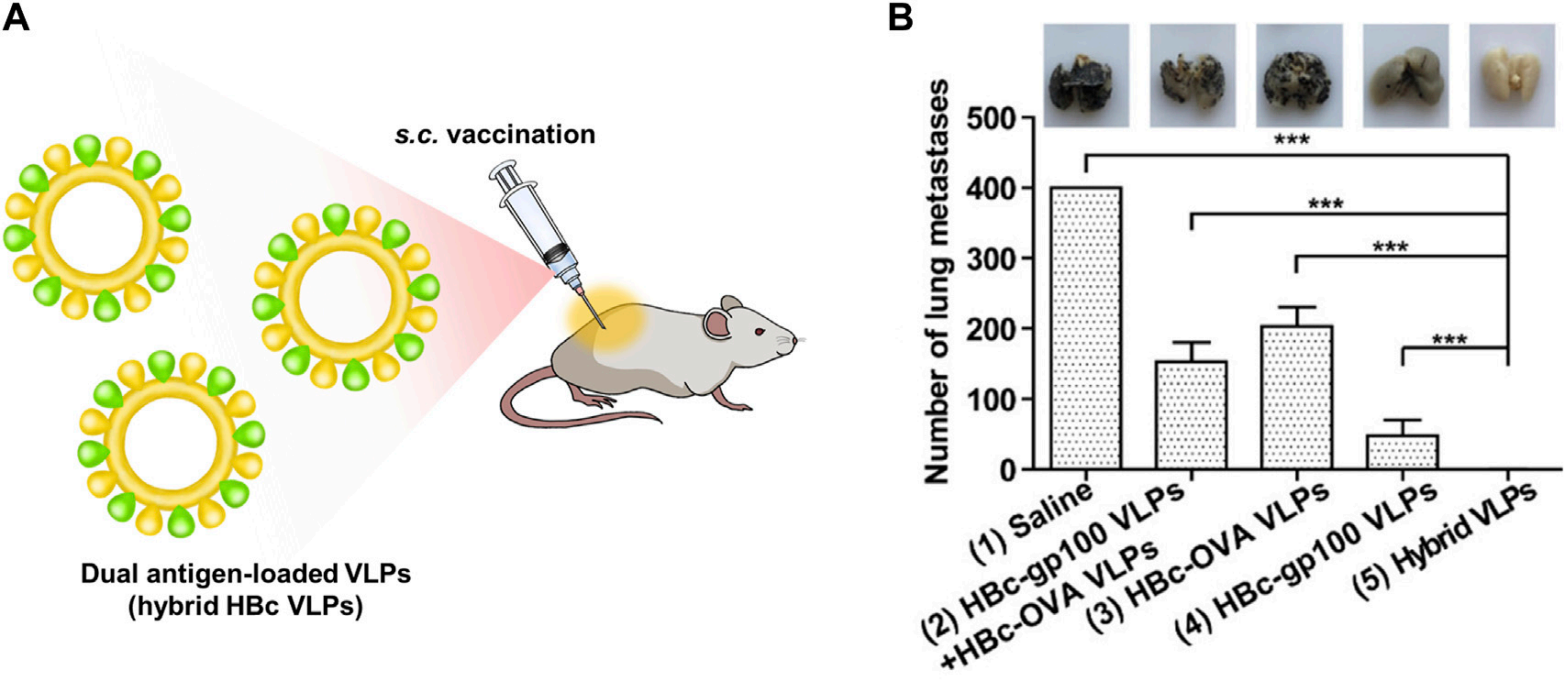
**(A)** Schematic diagram of dual antigen-loaded VLPs (hybrid HBc VLPs) for cancer immunotherapy. **(B)** Cancer immunotherapy efficiency of hybrid HBc VLPs in a lung metastatic tumor model. Reprinted with permission from Cheng et al. (2020). Copyright (2020) American Chemical Society.

Adjuvants assist cancer vaccines by triggering an immune response, however, they can induce an autoimmune response due to their toxicity (Kreutz et al., 2012). Therefore, the development of vaccines without adjuvants has been proposed. HBc VLPs displaying heterologous epitopes have been designed as an anti-cancer vaccine for melanoma (Cheng et al., 2020) (Figure 4). The HBc VLPs were genetically fused with OVA peptide and glycoprotein 100 (gp100). Gp100, one of the glycoproteins in melanocytes, is overexpressed in malignant tumors. Similar to the OVA peptide, the gp100 antigens induce an immune response by stimulating CTL activity melanoma (Keshavarz-Fathi and Razaei, 2019). The dual antigen-exposed HBc VLPs effectively inhibited tumor progression and metastasis. VLPs for chemotherapy were summarized in Table 1.

**TABLE 1:** Comparison of virus-like particles for drug delivery.

Virus-like particle^a^	Cargo material^b^	Treatment method	Loading method	Targeting method^c^	References
CCMV VLPs	DOX	Chemotherapy	Chemical conjugation	Active (FA)	Barwal et al., 2016
HBc VLPs	DOX	Chemotherapy	Chemical conjugation	Active (FA)	Biabanikhankahdani et al. (2018)
HBc VLPs	5-FA	Chemotherapy	Chemical conjugation	Active (CPP of EGFR)	Gan et al. (2020)
FMDV VLPs	DOX	Chemotherapy	Chemical conjugation	Active (RGD peptide)	Yan et al. (2017)
PhMV VLPs	Cisplatin	Chemotherapy	Chemical conjugation	Passive	Hu and Steinmetz, (2020b)
PhMV VLPs	DOX	Chemotherapy	Chemical conjugation	Passive	Hu and Steinmetz, (2020a)
HBc VLPs	DOX	Chemotherapy	Encapsulation	Passive	Biabanikhankahdani et al. (2016)
HBc VLPs	DOX	Chemotherapy	Chemical conjugation	Passive	Biabanikhankahdani et al. (2017)
HBc VLPs	DOX	Chemotherapy	Encapsulation	Active (RGD peptide)	Shan et al. (2018a)
CCMV VLPs	ODN1826	Gene therapy	Encapsulation	Passive	Cai et al. (2020)
CCMV VLPs	siRNA	Gene therapy	Encapsulation	Passive	Lam and Steinmetz (2019)
JCPyV VLPs	tK	Gene therapy	Encapsulation	Passive	Chao et al. (2018)
MS2 VLPs	MiR-122	Gene therapy	Encapsulation	Passive	Wang et al. (2016a)
HBc VLPs	siPLK1	Gene therapy	Encapsulation	Active (HER2 peptide)	Suffian et al. (2018)
HBc VLPs	siRNA for YAP, PTX	Chemotherapy, gene therapy	Encapsulation	Active (TGN peptide and RGD peptide)	Yang et al. (2020)
RHDV VLPs	TopIIα, Survivin	Vaccination	Genetic conjugation	Passive	Donaldson et al. (2017)
P22 VLPs	OVA_B_ peptide, OVA_T_ peptide	Vaccination	Genetic conjugation	Passive	Li et al. (2021)
Qβ VLP	Tf antigen, STn antigen	Vaccination	Chemical conjugation	Passive	Wu et al. (2019)
HBc VLPs	OVA peptide, gp100	Vaccination	Genetic conjugation	Passive	Cheng et al. (2020)

^a^ CCMV VLPs, cowpea chlorotic mottle virus virus-like particles; HBc VLPs, hepatitis B core protein virus-like particles; FMDV VLPs, foot-and mouth disease virus-like particles; PhMV VLPs, Physalis mottle virus virus-like particles; JCPyV VLPs, JC polyomavirus virus-like particles; MS2 VLPs, MS2 virus-like particles; RHDV, rabbit hemorrhagic disease virus; P22 VLPs, bacteriophage P22 VLPs; Qβ VLPs, bacteriophage Qβ VLPs.

^b^ DOX, doxorubicin; 5-FA, 5-fluorouracil-1-acetic acid; MiR-122, microRNA-122; ODN 1826, oligodeoxynucleotides 1826; OVA_B_ peptide, B epitopes of ovalbumin; OVA_T_ peptide; T epitopes of ovalbumin; PTX, paclitaxel; siPLK1, siRNA for polo-like kinase 1 gene; STn antigen, Sialyl thomsen-nouveau antigen; Tf antigen, Thomsen-Friedenreich antigen; tK, tymidine kinase suicide gene; TopIIα, Topoisomerase IIα; YAP, Yes-associated protein.

^c^ CPP, Cell-penetrating peptide; EGFR, epithelial growth factor receptor; FA, folic acid; HER2, Human epidermal growth factor receptor 2.

## 4 Bio-imaging

A delayed cancer diagnosis can increase the mortality risk (Neal, 2009; Torring et al., 2013; Neal et al., 2015); therefore, early cancer detection is crucial for successful cancer treatment. Bio-imaging of tissues and cells could be used for preclinical diagnosis, patient status monitoring, and the easy detection of diseased tissue during surgery. In recent years, bio-imaging technology using nanomaterials, including quantum dots, gold nanoparticles, silica nanoparticles, polymers, and VLPs, have been studied. In the following section, we discuss the development of bio-imaging technology using cancer-targeting VLPs.

### 4.1 Positron emission tomography (PET)

Positron emission tomography (PET) is a highly sensitive and non-invasive clinical modality. It is an important imaging tool for early cancer diagnosis and staging due to its high sensitivity and spatial resolution (Gambhir, 2002; Aanei et al., 2016). Various radioisotopes, such as ^18^F, ^64^Cu, and ^68^Ga, are used for PET; however, many of these have a short half-life and low specificity. Owing to their high biocompatibility and easy surface functionalization, VLPs have been used to avoid blood clearance and extend the half-life of radioisotopes by increasing the tracer size (Farkas et al., 2013; Shukla and Steinmetz, 2015).

Bacteriophage MS2 VLPs have been used as a carrier for the delivery of the radioisotope 18F (Hooker et al., 2008). The interior of MS2 VLPs was functionalized with ^18^F-fluorobenzaldehyde (FDG) *via* amino acid-specific conjugation. ^18^F-fluoride has a short half-life (110 min) and is quickly cleared by the blood circulatory system (Even-Sapir et al., 2007; Flexman et al., 2008). [^18^F]-MS2 VLPs were retained for 3 h *in vivo*, which is 1.6-fold longer than that of free ^18^F. Lambidis et al. (2022) utilized hepatitis E virus-like particles (HEVLPs) for the delivery of the radioisotope ^68^Ga. [^68^Ga]-DOTA was conjugated to the external lysine residues of HE VLPs. At 1 h post incubation, [^68^Ga]-DOTA-HE VLPs showed approximately a 2.5-fold higher cell internalization than that of free ^68^Ga. These results indicated that the increased size of VLP-based PET tracers extended the half-life and blood circulation time of radioisotopes, demonstrating the possibility of VLPs as carriers for PET.

PEG-functionalized MS2 VLPs have been developed to increase the blood circulation time of bacteriophage MS2-based PET tracers (Farkas et al., 2013). MS2 capsids were altered to contain *p*-aminophenylalanine and cysteine residues at positions T19 and N87. Their internal cysteine residues and external aniline moieties were conjugated to maleimide-DOTA-^64^Cu and PEG5k-aminophenol, respectively. These modified ^64^Cu-DOTA-MS2 VLPs were highly stable in serum and did not release a detectable level of ^64^Cu. ^64^Cu-DOTA-MS2 VLPs were present at a level three times higher than free ^64^Cu in the tumor site of MCF7cL18 cells-implanted mice model 24 h after injection. Furthermore, a 10-fold higher percent injected dose per gram of tissue (% ID/g) of ^64^Cu-DOTA-MS2 VLPs than that of free ^64^Cu was present in the blood. These data indicate that the VLPs improved the time ^64^Cu circulated in the blood in the form of ^64^Cu-DOTA-MS2, when compared to free ^64^Cu. However, a sufficient concentration of ^64^Cu-DOTA-MS2 VLP was not detected in the tumor site.

**FIGURE 5:**
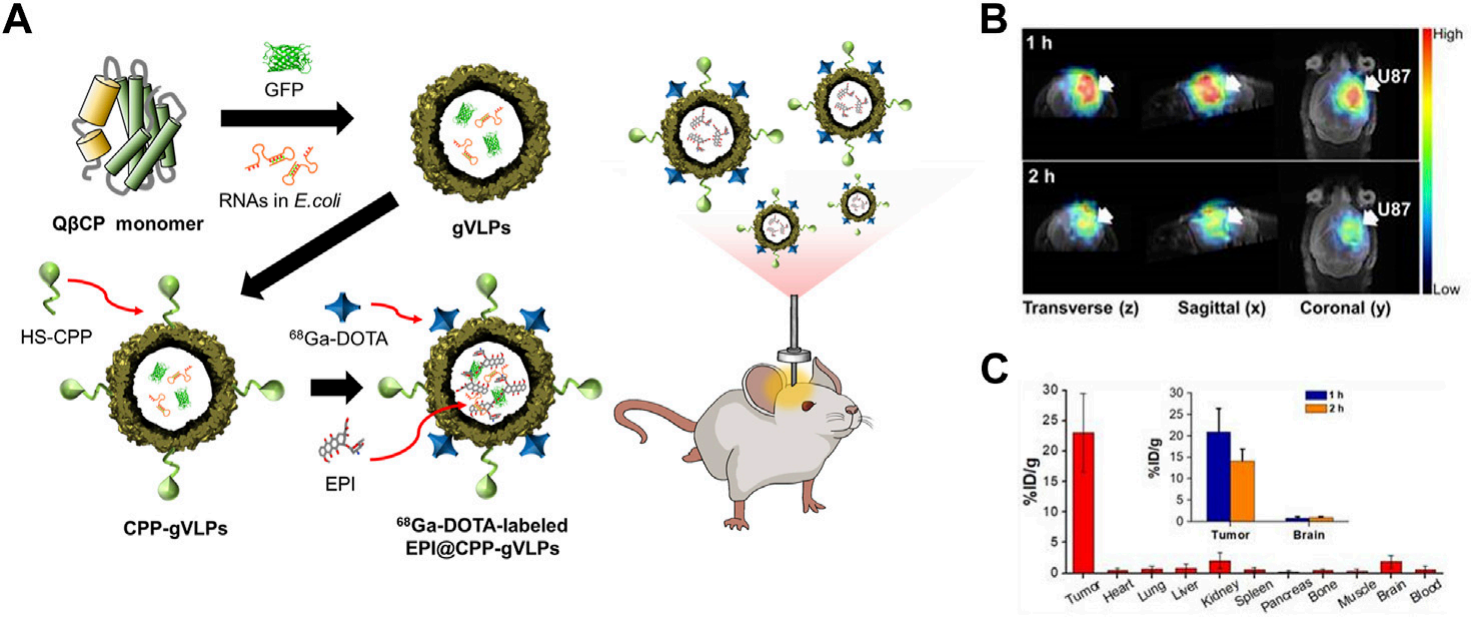
**(A)** Schematic diagram of the preparation of ^68^Ga-DOTA labeled EPI@CPP-gVLPs for bio-imaging brain tumors. **(B)** Representative micro-PET-MR images of U87-MGLu brain tumor mice using ^68^Ga-DOTA labeled EPI@CPP-gVLPs. **(C)** The organ distribution of ^68^Ga-DOTA labeled EPI@CPP-gVLPs (Pang et al., 2019a).

The ability of CPP-functionalized Qβ VLPs (CPP-Qβ-VLPs) to increase the cell internalization of VLP-based PET tracers has been explored (Pang et al., 2019a) (Figure 5). Cys-CPP and ^68^Ga-DOTA-NHS were conjugated to the exterior surface of Qβ VLP. CPP-Qβ-VLPs had a cell uptake efficiency of 72.9% in U87-MG cells after 5 h incubation, which was approximately twice as high as that of non-conjugated Qβ-VLPs. The high cellular uptake of ^68^Ga-DOTA-labeled CPP-Qβ-VLPs enabled the PET tracer to be detected in the tumor site.

The introduction of CPP and PEG improved the cell internalization of VLP-based PET tracers and increased the length of time they remained in the blood. However, these VLPs utilized the enhanced permeability and retention (EPR) effect-based passive targeting strategy, which is limited to tumors with low growth rates (Gambhir, 2002). Thus, it is crucial to develop a cancer-specific targeting strategy for clinical PET. Anti-human EGFR IgG1 monoclonal antibody was applied to ^64^Cu-DOTA-MS2 VLP to actively target EGFR-overexpressing HCC1954 breast cancer (Aanei et al., 2016); *p*-aminophenylalanine (*p*aF) unnatural amino acid was introduced at position 19 of the MS2 capsid proteins, followed by reacting with aminophenol-antibody conjugates. The specific targeting ability of ^64^Cu-DOTA-MS2-αEGFR was confirmed *in vitro* using flow cytometry and confocal live imaging with Oregon Green 488 (OG488)-labeled MS2-αEGFR as a control. MS2-αEGFR showed increased cellular internalization compared to the control in both EGFR-positive breast cell lines MDA-MB-231 and HCC 1954. Live imaging of a HCC1954-bearing breast cancer mouse model using PET revealed the *in vivo* biodistribution of ^64^Cu-DOTA-MS2-αEGFR. At 24 h after injection, the intensity of the signal was analyzed in the tumor site and a 4.9 tumor-to-muscle ratio (T/M) was detected, indicating a higher accumulation in the tumor region than in muscle.

### 4.2 Magnetic resonance imaging (MRI)

Magnetic resonance imaging (MRI) is an example of non-invasive imaging technology. In contrast to PET, MRI has excellent spatial resolution without the need for radioisotopes (Caravan, 2006). However, the slight difference in relaxation time between normal tissues and disease tissues results in low sensitivity, which prevents the accurate diagnosis of cancer in early stages (Na et al., 2009; Min et al., 2013; Qazi et al., 2013; Schwarz and Douglas, 2015; Usselman et al., 2015; Hu et al., 2019b). Thus, contrast agents are required to improve the accuracy of MRI by reducing relaxation times (Caravan et al., 1999; Caravan, 2006; Estelrich et al., 2015; Schwarz and Douglas, 2015; Shukla and Steinmetz, 2015). Nanoparticles are widely used as a scaffold for MRI contrast agents. Nevertheless, the currently used inorganic nanoparticle-based contrast agents are mostly untargeted, with low biocompatibility, and are difficult to load cargo (Estelrich et al., 2015). Owing to their capsular configuration with a high load capacity, considerable intracellular uptake ability, and easy functionalization, VLPs have been proposed as a scaffold for MRI contrast agents in order to overcome the limitations of conventional contrast agents (Mastrobattista et al., 2006; Gao et al., 2016). VLP-based MRI contrast agents showed significantly enhanced relaxivity both *in vitro* and *in vivo* compared to conventional contrast agents, demonstrating that VLP-based MRI contrast agents are potential candidates for cancer bioimaging.

Genetically modified HBc VLPs were chosen as a carrier for the iron oxide nanoparticle (Fe_3_O_4_-NTA-Ni^2+^) T2 MRI agent (Shen et al., 2015). During the disassembly and reassembly of HBc VLPs, HBc capsid proteins were successfully co-assembled into HBc VLPs with four different sizes of magnetic Fe_3_O_4_ nanoparticle (Fe_3_O_4_-HBc VLPs) owing to the interaction between the His_6_-tag on the N-terminal HBc capsid and the nickel-NTA chelate of Fe_3_O_4_ nanoparticles. Fe_3_O_4_-HBc VLPs exhibited three-fold higher cellular uptake efficiency than pure Fe_3_O_4_ nanoparticles in HeLa cells. Furthermore, the VLPs showed signal enhancement in HeLa cells with varying T2 relaxivity depending on the size of the encapsulated Fe_3_O_4_ nanoparticles (3.4, 6.1, and 11.7 nm).

A cancer-targeting ligand was applied to VLP-based MRI contrast agents to enable their target-specific delivery. Prostate cancer-specific Asp-Gly-Glu-Ala (DGEA) peptides-modified PhMV VLPs were developed with a multifunctional contrast agent (Cy5.5 and Gd^3+^(DOTA)) for both ultrahigh field magnetic resonance imaging (UHFMRI) and near-infrared fluorescence (NIFR) imaging (Hu et al., 2019a). The Cy5.5 and Gd^3+^(DOTA) imaging moieties were conjugated to the inner surface of PhMV VLPs using thiol-maleimide click chemistry. DGEA peptides were attached to the exterior of the PhMV VLPs *via* an NHS-PEG-maleimide linker. The synthesized Gd-Cy5.5-PhMV-mPEG NPs had a longitudinal relaxivity (r1) of 31.0 and 8.2 mM^−1^s^−1^ at 1.5 and 7 T, respectively, which is approximately 7-fold higher than that of a Gd^3+^(DOTA)-based commercial T1 MRI contrast agent. PC-3 tumor-bearing mouse models injected with Gd-Cy5.5-PhMV-mPEG NPs had an approximately 2-fold higher concentration of Gd^3+^ in the tumor site 240 h later than those injected with VLPs without DGEA ligands. This performance surpassed that of other VLP-based contrast agents (Lee et al., 2015; Pitek et al., 2016). These data demonstrated that the developed Gd-Cy5.5-PhMV-mPEG/DGEA enabled long-term tumor diagnosis with an enhanced T1 relaxation time and cancer-targeting ability.

Signal intensity in MRI is highly related to the concentration of the contrast agents (Shen et al., 2015). Most of the studied MRI imaging agents were loaded into the inner space of VLPs by either encapsulation or chemical conjugation. However, the concentration of encapsulated imaging agents can be estimated only by the inner capacity of the VLPs. Displaying imaging agents on the external surface of VLPs has been proposed in order to overcome this limitation. Bacteriophage M13 VLPs consist of 2,700 copies of major coat protein p8 and five copies of four different minor coat proteins (p9, p7, p6, and p3) (Li et al., 2010). Owing to their well-known structure and easy functionalization on the different coat proteins, bacteriophage M13 VLPs were used as a scaffold for contrast agents (Ghosh et al., 2012). Secreted protein acidic and rich in cysteine (SPARC)-binding peptide (SBP; a prostate cancer-specific peptide) and magnetic iron oxide particles were attached to the p3 and p8 capsid proteins, respectively (M13-SBP-magnetic nanoparticles (MNPs)). M13-SBP-MNP (58.7 mM^−1^s^−1^) performed better at 0.47 T *in vivo* than the current T2 MR contrast agents used clinically. Dark contrast was detected only in the SPARC-expressing prostate cancer, demonstrating that M13-SBP-MNP is able to specifically target prostate cancer. The signal-amplifying ability of M13 as a carrier was investigated using Alexa Fluor-modified peptide-functionalized MNPs (SBP-MNPs) and M13-SBP-MNPs. M13-SBP-MNPs exhibited an 11-fold higher fluorescence intensity than SBP-MNPs. These results indicated that M13-SBP-MNP could amplify MRI signal intensity owing to improved delivery efficiency of MNPs per carrier by cancer-specific targeting.

### 4.3 Fluorescence imaging

Fluorescence bio-imaging has been a promising non-invasive diagnostic method over the past few decades. It has several advantages, such as high sensitivity and easy modulation of the fluorescence signal by controlling the fluorescence probe. Near-infrared (NIR) fluorescence imaging has improved tissue penetration and reduced background signal compared to visible fluorescence imaging (Kosaka et al., 2009; Guerrero et al., 2015). Owing to these advantages, NIR fluorescence is well established in *in vivo* imaging. Cyanines are one of the most frequently used NIR fluorescent dyes with diverse clinical applications (Guerrero et al., 2017). However, their low stability and weak fluorescence emissions limit their application in bio-imaging (Samanta et al., 2010; Wu et al., 2013).

VLP-based nanocarriers have been proposed to improve the stability of fluorescent dyes. The fusogenic spike glycoprotein of vesicular stomatitis virus (VSV-G) was used as a protein nanocage for NIR bio-imaging (Bishnoi et al., 2021). VSV-G expressed in human embryonic kidney cell (HEK) 293T cells can be released in lipid-bound fusogenic nanovesicles of approximately 200 nm in size. Approximately 75% of indocyanine green dye (ICG) was successfully encapsulated in the internal core of VSV-G (NAVNs) by diffusion. In addition to the enhanced stability of ICG, these (VSV-G)-based NIR active viral nanoconstructs (NAVNs) showed a higher cellular uptake and delivery of ICG in HeLa cells than that of the free form of ICG. Furthermore, simian virus 40-derived VLP (SV40-VLP) was chosen as a carrier for the NIR-II fluorescent molecule CH1 (Min et al., 2021). SV40-VLPs produced in *Escherichia coli* were disassembled into virus particle (VP1) pentamers to encapsulate fluorescent molecules. During the reassembly process, successful encapsulation of CH1 (CH1-SV40) occurred owing to the non-covalent interactions between multiple amino acids on the VP1 pentamers and the NIR-II fluorescent molecule CH1. CH1-SV40 was highly stable under different conditions, particularly at low pH, suggesting that CH1-SV40 is suitable for *in vivo* bio-imaging of cancer cells with a slightly acidic extracellular microenvironment. The *in vivo* cancer imaging capability of CH1-SV40 was also demonstrated using a HeLa tumor-bearing mouse model; fluorescence signal remained at the tumor site for 5 h after the intra-tumor injection.

Plant virus-based VLPs, such as CCMV VLPs, have also been proposed as bio-imaging carriers. The CCMV shell comprises 180 identical subunits and can be disassembled into 90 capsid protein (CP) dimers *in vitro*. CP dimers can be reassembled into smaller structures than native CCMV, depending on the pH or ionic strength. Owing to this unique characteristic, CCMV is an attractive protein cage for various cargos (Wu et al., 2021). Viral RNA-removed CCMV CP was co-assembled with QD (Tagit et al., 2017). The assembly of CP dimers involves both CP-CP and CP-RNA interactions (Beren et al., 2020). Negatively charged QD replaced the viral RNA and interacted with CP dimers to drive the assembly. The cellular uptake of formed T = 1 and T = 3 QD/VLP nanoparticles was evaluated *in vitro*. Their fluorescence signals were retained in macrophages and HeLa cells for more than 18 h after incubation. Furthermore, QD/VLP nanoparticles resulted in no significant decrease in cell viability.

Brome mosaic virus (BMV), an icosahedral RNA plant virus belonging to the *Bromoviridae* family (Jung et al., 2011), can self-assemble and be stabilized by electrostatic interactions between the positively charged N-terminal of the coat protein and negatively charged RNA. This property enabled the construction of BMV VLP-based bio-imaging agents, called optical viral ghosts (OVGs), by replacing the viral RNA with negatively charged ICG (Guerrero et al., 2015; Jung et al., 2022). The developed OVGs showed potential as bio-imaging agents for detecting cancer cells. However, the low fluorescence intensity of ICG itself remains a drawback. To enhance the fluorescence quantum yield of ICG, brominated cyanine 106 *N*-hydroxysuccinimide (BrCy106-NHS) was applied to the OVGs (Guerrero et al., 2017). Bromine (Br) was placed in the aromatic rings of ICG to form BrCy106-NHS. Free BrCy106-NHS had increased stability and a 57-fold increased fluorescence intensity compared to those of the free form of ICG. Importantly, BrCy106-NHS-OVGs exhibited a 44-fold higher fluorescence emission than ICG-OVGs *in vivo* in ovarian cancer animal models. However, OVG-based bio-imaging agents displayed a low fluorescent dye encapsulation efficiency. Different NIR fluorescent dyes and encapsulation methods can affect the encapsulation efficiency, thus influencing the fluorescence signals. Furthermore, ICG has a self-quenching effect by aggregation; therefore, encapsulation methods may limit its application in fluorescence imaging.

**FIGURE 6:**
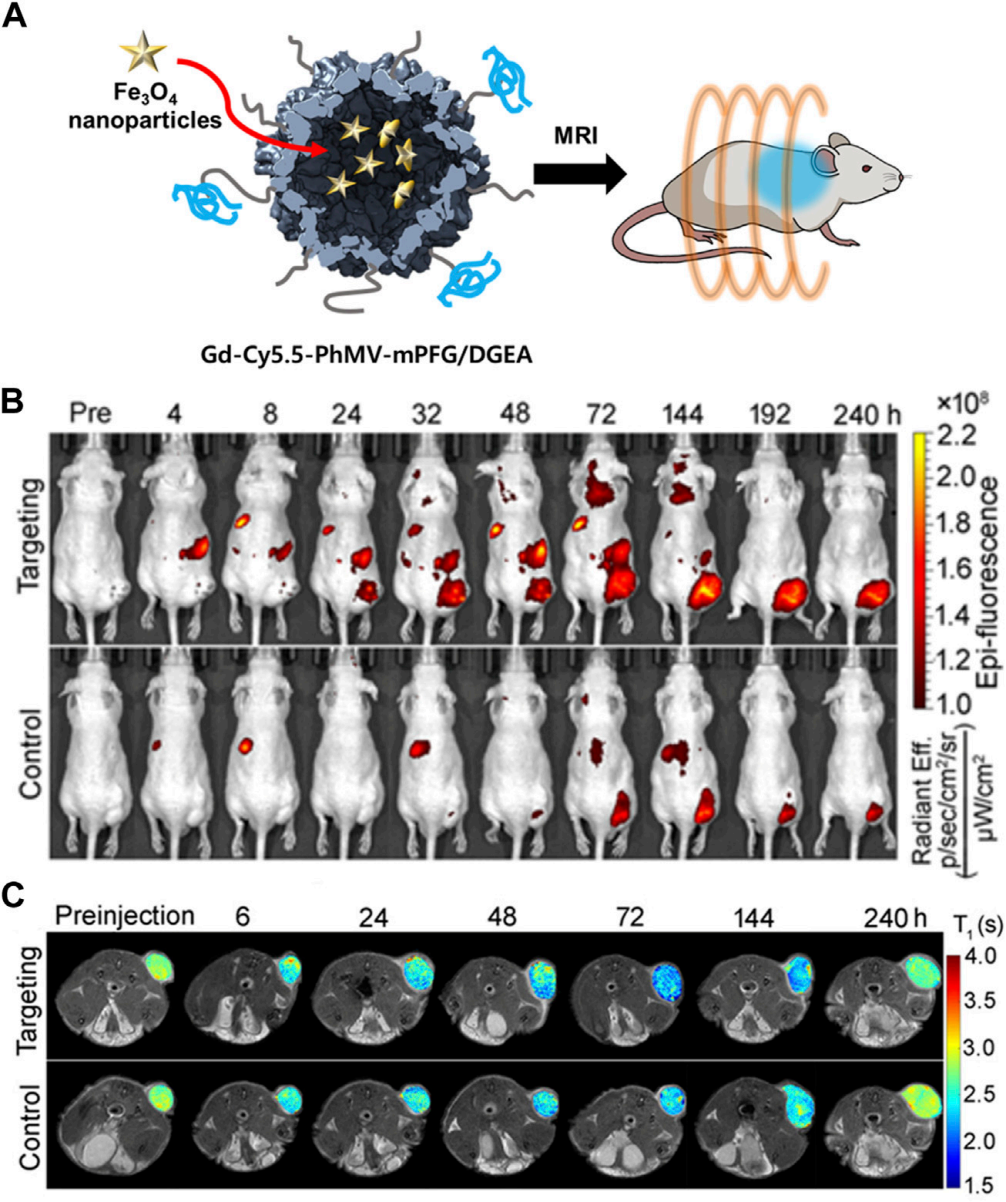
**(A)** Schematic illustration of Gd-Cy5.5-PhMV-mPEG NPs for cancer imaging. **(B)** *In vivo* NIR fluorescence images of PC-3 prostate tumors in athymic nude mice after the intravenous injection of Gd-Cy5.5-PhMV-DGEA. **(C)** *In vivo* T1-map of PC-3 prostate tumors in athymic nude mice after the intravenous injection of Gd-Cy5.5-PhMV-DGEA. Reprinted with permission from Hu et al. (2019a). Copyright (2019) American Chemical Society.

In addition to encapsulation, a fluorescent dye can be specifically labeled on the interior or exterior surface of VLPs by chemical conjugation whilst maintaining an appropriate distance and concentration to minimize the quenching effect (Wu et al., 2005). The cyanine dyes Cy5 and Cy5.5 were chemically conjugated to the interior and exterior of PhMV, respectively (Masarapu et al., 2017). Sulfo-Cy5-NHS esters and Cy5.5-maleimide were conjugated to the lysine and cysteine residues of PhMV, respectively (PhMV-K_E_-Cy5 and PhMV-C_I_-Cy5.5). The conjugation condition was optimized by changing the reaction time and the molar ratios of the dyes to PhMV to minimize self-quenching. By using amine-specific chemical conjugation, the distance between Cy5 molecules conjugated to the surface was approximately 2–4 nm. Only one Cy5.5 was bound by thiol-specific conjugation. PhMV-K_E_-Cy5 and PhMV-C_I_-Cy5.5 exhibited high cellular uptake efficiencies in ovarian, breast, and prostate cancer cells. Therefore, PhMV-based VLPs are a potential carrier for cancer bio-imaging due to their high cellular uptake efficiency and spatial distribution of fluorescence probes. Hu et al. (2019a) conjugated PhMV to a multifunctional imaging agent (Figure 6). The fluorescent probe Cy5.5 and MRI agent Gd^3+^ were conjugated to the interior surface of PhMV by a thiol-maleimide reaction. The outer surface was modified by PEGylation using mPEG-NHS or maleimide-mPEG-NHS (Gd-Cy5.5-PhMV-mPEG NPs). DGEA peptides were also conjugated to the PEG terminal end of Gd-Cy5.5-PhMV-mPEGs in order to target prostate cancer cells (Gd-Cy5.5-PhMV-mPEG/DGEA). The cellular uptake of Gd-Cy5.5-PhMV-mPEG/DGEA was evaluated in the α2β1 integrin-overexpressing prostate cancer cell line PC-3. The fluorescence image showed strong signal intensity in PC-3 cells, suggesting that Gd-Cy5.5-PhMV-mPEG/DGEA might be able to specifically target prostate cancer cells. Additionally, fluorescent signal from the conjugated Cy5.5 was detected in some CD45-and CD68-expressing immune cells, indicating that Gd-Cy5.5-PhMV-mPEG/DGEA could be delivered by immune cells to the tumor region, resulting in a prolonged signal in the tumor.

**FIGURE 7:**
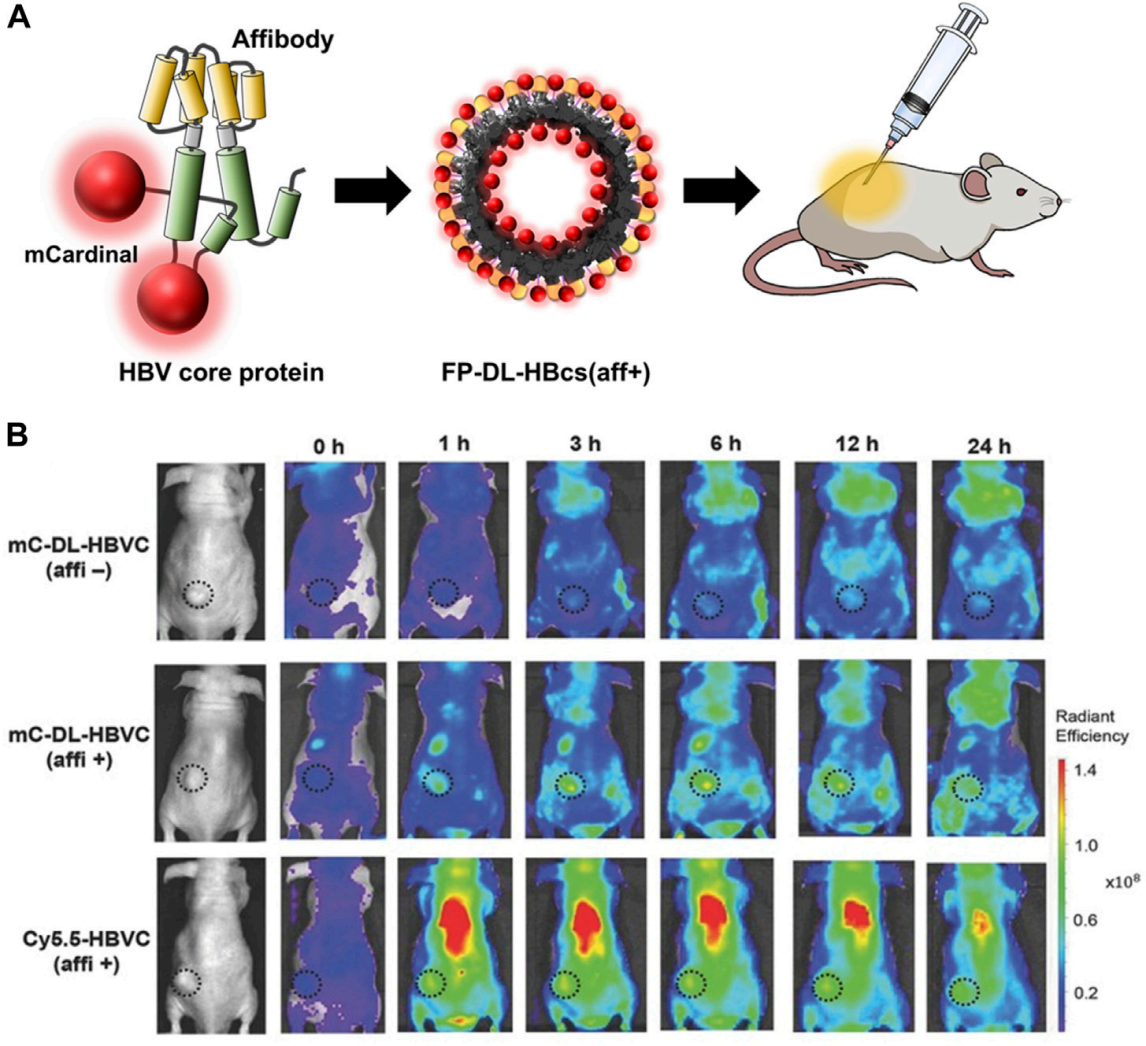
**(A)** Schematic diagram of the preparation of FP-DL-HBcs for bio-imaging of cancer cells. **(B)** NIR fluorescence images of MDA-MB-468 tumor-bearing mice after the intravenous injection of FD-DL-HBcs (Kim et al., 2017).

Chemical conjugation is a way to label imaging probes with a suitable spatial arrangement to reduce the quenching effect, however, it is not easy to control the site-specific conjugation precisely. Genetic conjugation has been proposed to overcome this limitation. Three types of fluorescent protein-inserted hepatitis B virus capsid proteins (FP-HBcs) have been developed (Figure 7) (Kim et al., 2017). The fluorescent proteins (FPs) were located in the following: 1) the internal layer, 2) the outer surface, and 3) both the internal and external layers of HBcs. Each FP was inserted at specific sites, and the self-quenching of FPs was controlled by varying the length of the flexible peptide linkers. All the developed FP-HBcs showed high fluorescent signals and photostability, particularly the double-layered FP-HBcs (FP-DL-HBcs). To further exploit the FP-HBcs for tumor imaging, cancer cell receptor-binding peptides (affibodies) were inserted into the outer surface of the FP-HBcs. The affibodies enabled the HBc to actively target human epidermal growth factor receptor 1 (EGFR) that is overexpressed in cancer cells, while decreasing immunogenicity. mCardinal FP-DL-HBcs with affibodies (mC-DL-HBcs [aff+]) were successfully internalized into EGFR-overexpressing tumor cells and retained a higher fluorescence intensity than mCardinal FP. Notably, mC-DL-HBc [aff+] could be detected in the tumors of live mice with far less accumulation in the liver compared to that of fluorescent Cy5.5. VLPs for bio-imaging were summarized in Table 2.

**TABLE 2:** Comparison of virus-like particles for bio-imaging.

Virus-like particle^a^	Cargo material^b^	Loading method	Imaging method^c^	References
MS2 VLP	FDG	Chemical conjugation	PET	Hooker et al. (2008)
HEVLPs	^68^Ga-DOTA	Chemical conjugation	PET	Lambidis et al. (2022)
MS2 VLPs	^64^Cu-DOTA	Chemical conjugation	PET	Farkas et al. (2013)
Qβ VLPs	^68^Ga-DOTA	Chemical conjugation	PET	Pang et al. (2019a)
MS2 VLPs	^64^Cu-DOTA	Chemical conjugation	PET	Aanei et al. (2016)
HBc VLPs	Fe_3_O_4_-NTA-Ni^2+^	Encapsulation	MRI	Shen et al. (2015)
PhMV VLPs	Gd^3+^-DOTA, Cy5.5	Chemical conjugation	Fluorescent, UHFMRI	Hu et al. (2019a)
M13 VLPs	MNPs	Electrostatic interaction	MRI	Ghosh et al. (2012)
VSV-G VLPs	ICG	Encapsulation	Fluorescent	Bishnoi et al. (2021)
SV40-VLPs	CH1	Encapsulation	Fluorescent	Min et al. (2021)
CCMV VLPs	QDs	Encapsulation	Fluorescent	Tagit et al. (2017)
BMV VLPs	BrCy106	Encapsulation	Fluorescent	Guerrero et al. (2017)
PhMV VLPs	Cy5, Cy5.5	Chemical conjugation	Fluorescent	Masarapu et al. (2017)
HBc VLPs	eGFP, mCardinal	Genetic modification	Fluorescent	Kim et al. (2017)

^a^ BMV VLPs, brome mosaic virus virus-like particles; CCMV VLPs, cowpea chlorotic mottle virus virus-like particles; HBc VLPs, hepatitis B core protein virus-like particles; HEVLPs, hepatitis E virus-like particles; MS2 VLPs, bacteriophage Emesvirus zinderi MS2 virus-like particles; PhMV VLPs, Physalis mottle virus virus-like particles; Qβ VLPs, bacteriophage Qubevirus durum VLPs; SV40 VLPs, Simian virus 40 virus-like particles; VSV-G VLPs, Vesicular stomatitis virus virus-like particles.

^b^ ByCy106, brominated cyanine 106; Cy, cyanine; DOTA, tetraazacyclododecane tetraacetic acid; eGFP, enhanced green fluorescent protein; FDG, 18F-fluorobenzaldehyde. ICG, indocyanine green; MNPs, Magnetic nanoparticles; QDs, Quantum dots.

^c^ MRI, magnetic resonance imaging; PET, positron emission tomography; UHFMRI, ultrahigh field magnetic resonance imaging.

## 5 Theragnosis

Theragnosis is an effective strategy for the management of specific diseases and combines diagnosis and therapy (Lee et al., 2012; Ryu et al., 2012; Doan et al., 2021; Dessale et al., 2022). Generally, theragnosis requires non-invasive imaging methods, including fluorescence, photoacoustic imaging (PAI), positron emission tomography (PET), and MRI. By combining treatment with non-invasive imaging methods, theragnosis enables an early and precise diagnosis, avoiding unnecessary treatment and reducing the related side effects.

Early research in theragnosis technology focused on combining therapeutic and imaging agents in one delivery system while enabling them to carry out their roles individually (Lim et al., 2015). GFP-Qβ VLPs have been used as carriers for cancer theragnosis (Pang et al., 2019a). GFP-Qβ VLPs were prepared by expressing coat proteins and GFP simultaneously. Cys-CPP was conjugated to the surface of GFP-Qβ VLPs using sulfosuccinimidyl 4-(N-maleimidomethyl) cyclohexane-1carboxylate (Sulfo-SMCC) to enable the carriers to cross the blood-brain barrier (BBB) (Tang et al., 2019). ^68^Ga and epirubicin (EPI) were used for theragnosis as imaging and therapy agents, respectively. EPI was loaded into the GFP-Qβ VLPs by diffusion, and 57.3% of EPI was released in slightly acidic conditions. This result suggests that EPI could be released in the acidic tumor microenvironment. Owing to its slow release, it was also possible to minimize the damage to normal tissue. The ^68^Ga radioisotope was conjugated to the exterior of the VLPs using an amine-reactive NHS-ester. The biodistribution of the VLPs in a glioblastoma animal model was analyzed using both PET/CT and fluorescence imaging. After 2 h post-injection, the VLPs remained in the tumor region with minimal diffusion into surrounding normal tissues. Moreover, the tumor was eradicated after 8 days with 2 doses of the VLPs, in contrast to mice treated with free EPI that died after 3 days. These results indicated that the developed VLP carrier was highly efficient at delivering its cargo in a localized manner and was less toxic than EPI. Pang et al. (2019b) co-expressed and assembled GFP-Qβ coat proteins with c-MET targeted miRNA (RNAic-MET) in *E. coli*. RNAic-MET is known to induce the degradation of c-MET mRNA, which is associated with tumor cell proliferation and survival (Christensen et al., 2005; Zhan et al., 2020). In order to specifically target brain tumors, CPP and an apolipoprotein E peptide (ApoEP) were attached to the external surface of GFP-Qβ VLPs-RNAic-MET (dP@gVLP/RNAic-MET). The fluorescent bioimaging showed a 3.2-fold enhanced fluorescent signal intensity in GBM U87 cells after 24 h incubation compared to that of the non-targeted control. As a result of the RNAi being released from the dP@gVLP carrier, c-MET expression was reduced in U87 glioblastoma cells. In the U87 tumor-bearing animal model, dP@gVLP/RNAic-MET was found to penetrate the BBB with high efficiency and cause significant tumor growth suppression, particularly when paired with temozolomide (TMZ).

Recently, light-based treatment, including photothermal therapy (PTT) and photodynamic therapy (PDT), has been introduced to simplify theragnostic particle synthesis (Ryu et al., 2012). PTT is a photo-absorbent-based treatment method that kills cells using the heat generated by exposing them to near-infrared (NIR) light (Nomura et al., 2020). PDT is a treatment method involving the use of a photosensitizer (PS) that, when activated by light, generates reactive oxygen species (ROS), which kill tumor cells (Gunaydin et al., 2021).

The NIR dye IR-780 enables the combination of PTT and PDT because it can be used as a photosensitizer for both PTT and PDT. However, IR-780 iodide is lipophilic, which limits its practical use and *in vivo* efficiency (Wang et al., 2016b; Cai et al., 2021). HBc VLPs were proposed as a carrier for IR-780 iodide (Lu et al., 2021). IR-780 iodide was loaded into HBc VLPs using a thermal-triggered encapsulation at 60°C (HBc VLP-IR780). HBc VLP-IR-780 accumulated significantly and was maintained in the tumor tissue more than in other major organs, which might be due to the RGD inserted on the outer surface of the HBc VLPs. The encapsulated IR-780 in HBc VLPs exhibited higher photostability and photothermal conversion efficiency and produced more ROS than the free form of the IR-780 iodide. HBc VLP-IR780 exhibited significant anti-tumor effects due to the synergy of the tumor-targeting HBc VLPs and IR-780 iodide enabling both PDT and PTT. However, this method relies on the EPR effect to target cancer cells.

**FIGURE 8:**
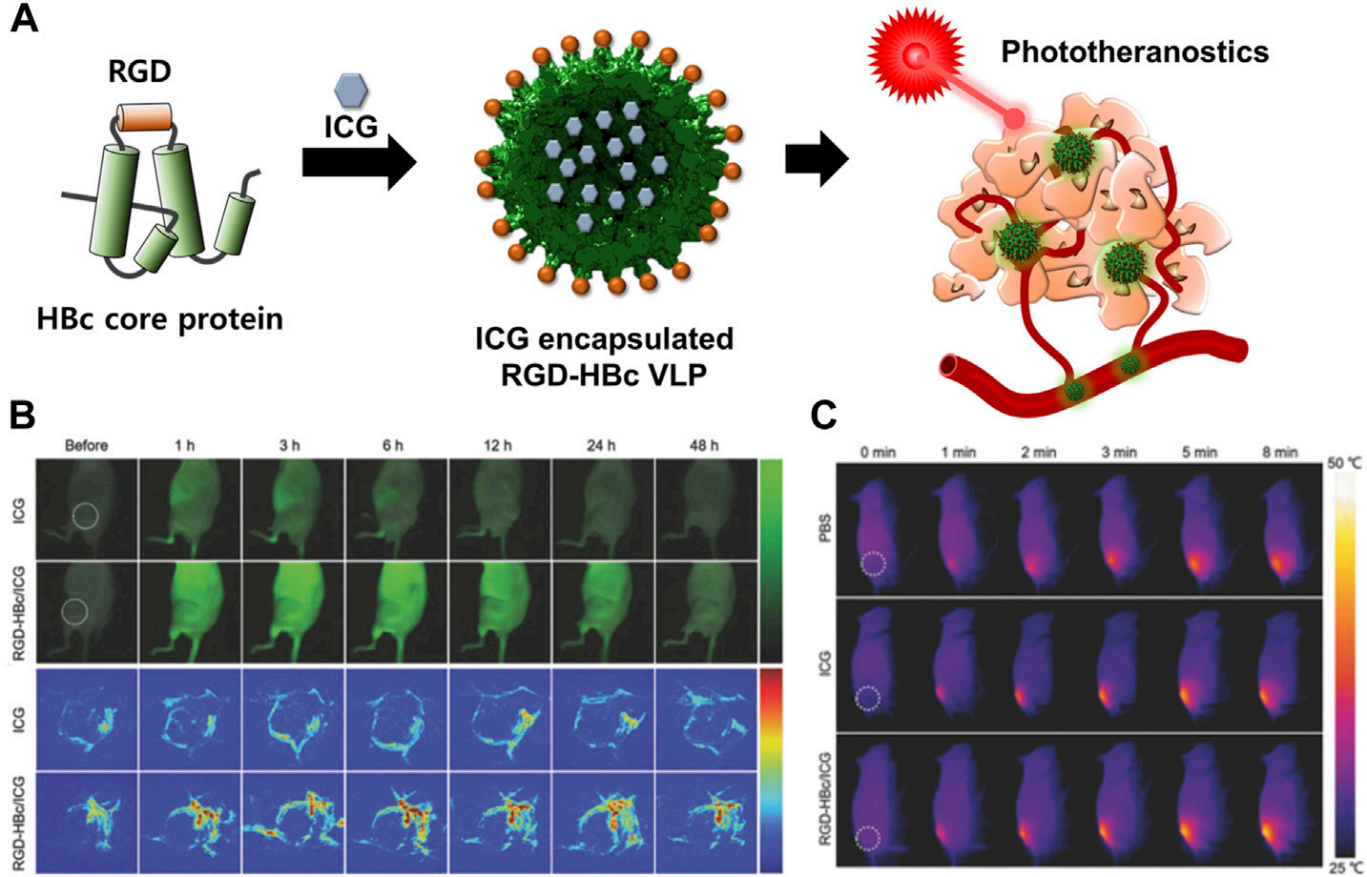
**(A)** Schematic illustration of the preparation and mechanism of action of RGD-HBc/ICG VLPs for cancer therapy and diagnosis. **(B)** *In vivo* fluorescence images of U87MG tumor-bearing mice and photoacoustic images of tumor blood vessels after the intravenous injection of RGD-HBc/ICG VLPs. **(C)** *In vivo* photothermal response of U87MG tumor-bearing mice after tail vein injection of RGD-HBc/ICG VLPs and laser exposure. Reprinted with permission from Shan et al. (2018b). Copyright (2018) Wiley-VCH GmbH.

ICG can be used for NIFR and PAT imaging as well as for both PTT and PDT (Sheng et al., 2013). However, the dye has some limitations, including instability, self-aggregation in aqueous solution, and non-specific interaction with proteins leading to its rapid elimination from the body. In addition, the dye cannot be targeted to specific cells (Yaseen et al., 2007; Kirchherr et al., 2009). HBc VLP was used as a carrier to encapsulate ICG (Figure 8) (Shan et al., 2018b). The dye was loaded into the inner space of RGD-inserted HBc VLPs using the disassembly and reassembly method and electrostatic interaction. Encapsulated ICG in RGD-HBc VLPs (RGD-HBc/ICG VLP) exhibited high aqueous stability and photothermal conversion ability and produced more ROS than free ICG. RGD-HBc VLPs also showed a prolonged circulation time and profound tumor-specific accumulation compared to free ICG. These enhanced properties enabled more accurate and sensitive imaging of human glioblastoma U87MG and longer lasting therapeutic effects than that using free ICG. These results demonstrated that HBc VLPs could maximize the advantages of medicinal ICG while overcoming its limitations.

**FIGURE 9:**
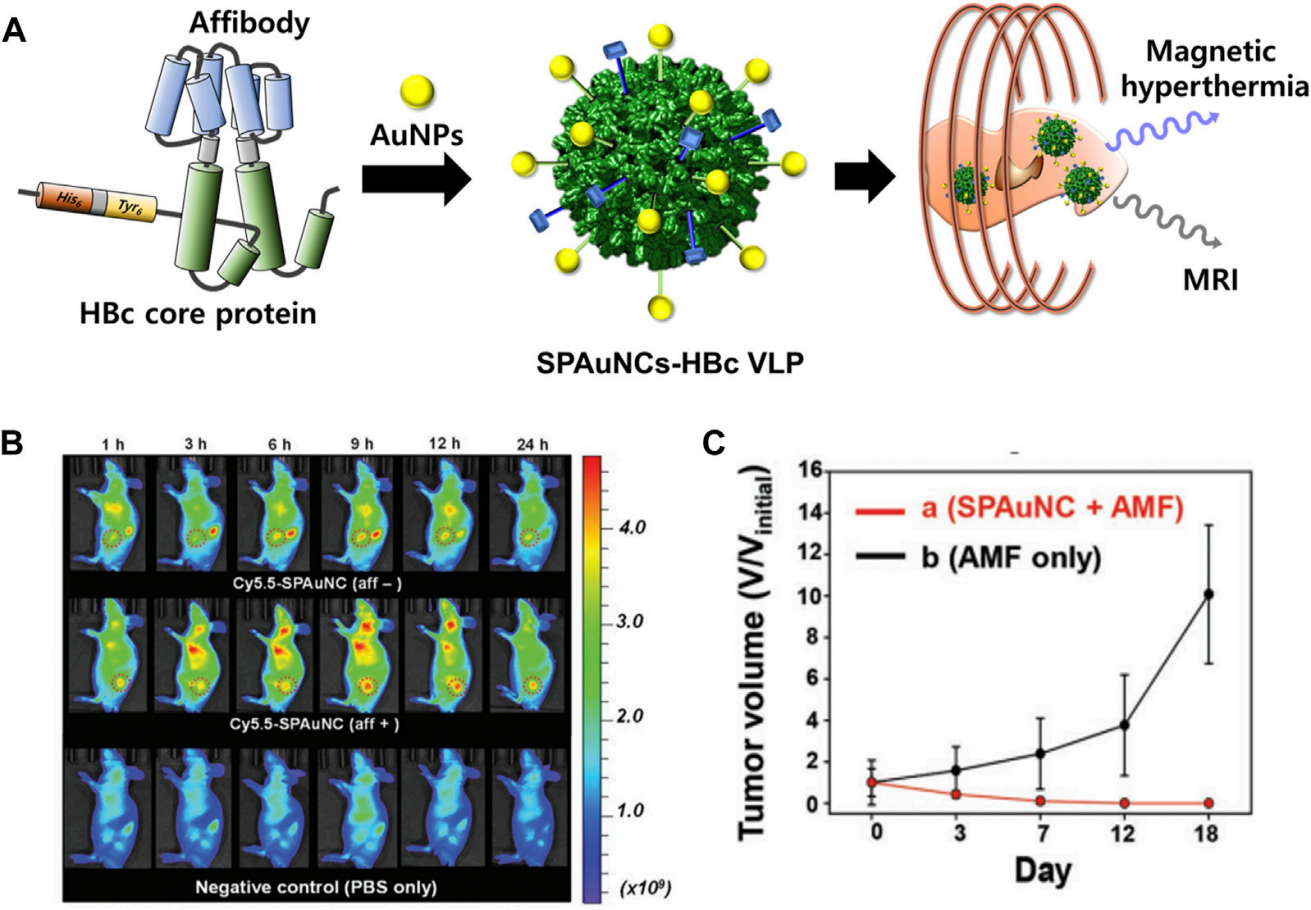
**(A)** Schematic illustration of the preparation of SPAuNCs for cancer theragnosis. **(B)** NIR fluorescence images of mice bearing a subcutaneous MDA-MB-468 tumor after the intravenous injection of SPAuNCs. **(C)** Cancer treatment efficiency of SPAuNCs. Reprinted with permission from Kwon et al. (2017). Copyright (2017) Wiley-VCH GmH.

In addition, superparamagnetic gold-nanoparticle clusters (SPAuNCs) have been utilized as MRI agents in HBc VLPs (SPAuNCs-HBc VLPs) (Figure 9) (Kwon et al., 2017). HBc capsid proteins were genetically modified by inserting His6-spacer peptide-Tyr6 at the N-terminus and replacing Pro79Ala80 with the tandem repeat of the affibody peptide for human epidermal growth factor receptor I (EGFR), which is overexpressed in various tumor cells. SPAuNCs, composed of tiny gold nanoparticles less than 2 nm, were produced by reducing gold ions at the N-terminal Tyr6 of the modified HBc VLPs. The SPAuNCs-HBc VLPs enabled specific tumor targeting, T2-weighted MRI, and magnetic hyperthermia therapy in the MDA-MB-468 tumor-bearing mice.

To achieve improved treatment efficacy, VLPs have been suggested as a carrier for dual-modal therapy and diagnosis. HBc VLPs were used as a carrier to encapsulate methotrexate (MTX)-conjugated iron oxide (Fe_3_O_4_) nanoparticles (Zhang et al., 2018b). These nanoparticles were loaded into the inner space of HBc VLPs using disassembly/reassembly method. Fe_3_O_4_-MTX@HBc VLP-treated murine breast cancer 4T1 cells exhibited 20% more cytotoxicity than untreated 4T1 cells. When exposed to light, the particles resulted in 60% more cytotoxicity than non-irradiated particles. These results suggest that tumor cells could be eliminated through the synergistic effect of PTT and chemotherapy. A T2-weighted MRI showed that the surrounding cancer cells were darker than the normal cells at 30 min after injection of Fe_3_O_4_-MTX@HBc VLPs into 4T1 tumor-bearing BALB/c mice. Fe_3_O_4_-MTX@HBc VLPs also resulted in reduced tumor growth in the tumor-bearing mice compared to the untreated group. However, Fe_3_O_4_-MTX@HBc VLPs accumulated significantly in both normal and cancer cells. VLPs for theragnosis were summarized in Table 3.

**TABLE 3:** Comparison of virus-like particles for theragnosis.

Virus-like particle^a^	Cargo material^b^	Loading method	Theragnosis method^c^	Targeting method^d^	References
Qβ VLPs	GFP, ^68^Ga-DOTA, EPI	Encapsulation	Fluorescence imaging, PET/CT	Active (CPP peptide)	Pang et al. (2019a)
Qβ VLPs	GFP, RNAi_c-MET_	Encapsulation	Fluorescence imaging, Chemotherapy	Active (CPP peptide, APoEP)	Pang et al. (2019b)
HBc VLPs	IR-780 iodide	Encapsulation	Near infrared fluorescence	Active (RGD peptide)	Lu et al. (2021)
			PTT, PDT		
HBc VLPs	ICG	Encapsulation	NIFR, PAT	Active (RGD peptide)	Shan et al. (2018b)
			PTT, PDT		
HBc VLPs	SPAuNCs	Chemical conjugation	MRI	Active (EGFR affibody)	Kwon et al. (2017)
			Magnetic hyperthermia		
HBc VLPs	MTX, Fe_3_O_4_	Encapsulation	MRI	Passive	Zhang et al. (2018b)
			PTT, chemotherapy		

^a^ HBc VLPs, hepatitis B core protein virus-like particles; Qβ VLPs, bacteriophage Qubevirus durum virus-like particles.

^b^ DOTA, tetraazacyclododecane tetraacetic acid; EPI, epirubicin; GFP, green fluorescent protein; ICG, indocyanine green; MTX, methotrexate; RNAi_c-MET_, miRNA for c-MET; SPAuNCs, superparamagnetic gold nanoparticle clusters.

^c^ NIFR, near-infrared fluorescence; MRI, magnetic resonance imaging; PTT, photothermal therapy; PDT, photodynamic therapy.

^d^ APoEP, apolipoprotein E peptide; EGFR, epidermal growth factor receptor.

## 6 Conclusion

In this review, we focused on current developments in the successful application of engineered VLPs in cancer diagnosis and therapy. However, most VLPs have been delivered to tumors through passive targeting based on the EPR effect. Only a few studies have used the RGD peptide or folic acid to actively deliver VLPs. There are some limitations to the practical application of VLPs in cancer therapy and diagnosis, and currently there are only a few VLPs being considered for theragnosis. Research on ensuring the structural stability of VLPs is ongoing. Continuous developments in VLP-based therapy and diagnosis strategies will be needed to overcome these limitations.
